# Expression of Melanocortin-4 Receptor mRNA in Male Rat Hypothalamus During Chronic Stress

**Published:** 2015

**Authors:** Maryam Karami Kheirabad, Bahia Namavar Jahromi, Amin Tamadon, Amin Ramezani, Somayeh Ahmadloo, Fatemeh Sabet Sarvestan, Omid Koohi-Hosseinabadi

**Affiliations:** 1 *Department of Basic Sciences, Azad University, Gachsaran Branch, Gachsaran, Iran.*; 2 *Infertility Research Center, Department of OB-GYN, School of Medicine, Shiraz University of Medical Sciences, Shiraz, Iran.*; 3 *Transgenic Technology Research Center, Shiraz University of Medical Sciences, Shiraz, Iran.*; 4 *Department of Medical Biotechnology, School of Advanced Medical Sciences and Technology, Shiraz University of Medical Sciences, Shiraz, Iran.*; 5 *Institute of Cancer Research, Shiraz University of Medical Sciences, Shiraz, Iran.*; 6 *Laboratory Animal Center, Shiraz University of Medical Sciences, Shiraz, Iran.*

**Keywords:** Chronic stress, melanocortin 4 receptor (MC4R), hypothalamus, rats

## Abstract

The effects of chronic stress and glucocorticoids receptor antagonist (RU486) on expression of melanocortin 4 receptor (MC4R) mRNA in arcuate nucleus (ARC) of male rats were evaluated. In this study, adult male Sprague Dawley rats were placed into four groups (n=6/group); stress, RU486, stress/RU486, and control groups. In stress group, the rats were restrained, 1 h/day, for 12 days. In RU486 group, the rats were injected RU486 for 12 days. In stress/RU486 group, the rats were injected RU486 1 h before the stress process for 12 days. Relative expression of MC4R mRNA was determined using real-time PCR. Relative expression of MC4R mRNA in the stress group was higher than that of the control rats (P<0.05). Relative expressions of MC4R mRNA were not different between the stress, RU486 and stress/RU486 groups (P>0.05). Chronic restraint stress causes increase in mRNA expression of MC4R in ARC and blockade of glucocorticoid receptors has no effect on this up-regulation.

The melanocortin-4 receptor (MC4R) is a member of G-protein coupled receptor super-family, expressed predominantly in the central nervous system (1) and is essential for feeding and energy balance (2, 3). α–melanocyte- stimulating hormone (α-MSH), endogenous MC4R agonist, agouti-related peptide (Agrp), and endogenous MC4R antagonist act on MC4R and plays a critical role in energy homeostasis (4, 5). MC4R is highly expressed in the paraventricular (PVN) and arcuate (ARC) nuclei of the hypothalamus, the main areas of the brain that directly regulate the activity of stress axis (6, 7), indicating a potential role for MC4R in stress system.

MC4R co- expressed with corticotrophin releasing hormone (CRH) containing neurons in hypothalamus (8) and central melanocortin increase adrenocorticotropic hormone (ACTH) plasma concentration (9, 10). Central administration of MC4R agonist increases anxiety behaviors (11). The stress response leads to the production of glucocorticoids via activation of a cascade conducted through the hypothalamic-pituitary- adrenal (HPA) axis. The effects of long-term stress exposure are numerous and complex. Some of these adverse effects on health are increased cardiovascular diseases, increased prevalence of obesity, depression and anxiety disorder (12).

Although there are indications that MC4R is involved in the stress systemit is not clear if glucocorticoids have effects on expression of MC4R in stress. The present study considers the way chronic restraint stress modulates hypothal-amic MC4R expression using RU486, a glucoco-rticoids receptor antagonist in adult male rats.

## Materials and methods

**Animals**

Twenty-four adult male Sprague- Dawley rats with mean and standard deviation (SD) weight of 236.8± 23.5 g were housed under controlled temperature 23± 1°C (mean± SD), 12 h light/dark cycle and 55± 5% (mean± SD) relative humidity in the L-laboratory animal center, Shiraz University of Medical Sciences, Iran. Animals were given free access to standard diet pellet and water *ad libitum* during experimental period. The experimental investigation was approved by Shiraz University of Medical Sciences Ethics Committee.

**Chronic stress induction**

The rats were randomly divided into four equal groups (n=6) including stress, RU486, stress/RU486, and control groups. In stress group, the rats were individually restrained for 1 hour session in plastic cylinders (20.5 × 8 × 6 cm) which had holes for ventilation and their extended tails. The cylinders were just large enough to allow rats of the size used to turn around easily. In RU486 group, the rats were injected subcutaneously with RU486 (2.5 mg/kg, 20 μl/rat; ab120356, Abcam Ltd, Cambridge, UK) for 12 days. In stress/RU486 group, the rats were injected subcutaneously with the same dose of RU486 1h before the stress process for 12 days. The control group rats were allowed to move freely in the laboratory rat cage type III and did not receive chronic stress and/or RU486.

**MC4R real-time PCR**

Five male rats were used as the control castrated group for real-time PCR. The rats were weighted and anaesthetized by an intraperitoneal injection of ketamine (100 mg/kg; Woerden, Netherlands) and xylazine (7 mg/kg; Alfazyne, Woerden, Netherlands) and castrated through ventral midline incision. Further procedures were carried out after a 2-week recovery period.

Brains of five groups of rats were immediately removed and the diencephalon was dissected out by an anterior coronal section, anterior to the optic chiasm, and a posterior coronal cut at the posterior border of the mammillary bodies. To separate ARC from AVPV, a third coronal cut was made through the middle of the optic tract, just rostral to infundibulum (13). The specimens consisted of ARC were stored in liquid nitrogen until further analysis.

Total RNA was extracted using the Tripure isolation reagent (Roche Life Science, Branford, CT). Briefly, the tissue (100 mg) was ground in liquid nitrogen, transferred to Tripure isolation reagent RNX-Plus buffer (1 mL) in an RNase-free microtube, mixed thoroughly, and kept at room temperature for 5 min. Chloroform (0.2 mL) was added to the slurry, mixed gently and incubated at room temperature for 15 min. The mixture was centrifuged at 12,000 × g (4 °C) for 20 min and the supernatant was transferred to another tube and precipitated with an equal volume of isopropanol for 15 min. The RNA pellet was washed with 75% ethanol and quickly dried and re-suspended in 50 µL RNase-free water. The integrity and quantity of RNA was checked by visual observation of 28S and 18S rRNA bands on a 1% agarose gel. The purified total RNA was quantified by Nano-Drop ND 1000 spectrophotometer (Nano-Drop Technologies, Wilmington, DE, USA). The DNase treatment was carried out using a DNase kit (Fermentas, St. Leon-Roth, Germany) according to the manufacturer’s instructions. The DNase-treated RNA was used for the first strand cDNA synthesis according to manufacturer’s instructions (Fermentas, St. Leon-Roth, Germany) in a 20 µL final volume. Primers were designed (Table 1) using Allele ID 7 software (Premier Biosoft International, Palo Alto, USA) for reference gene MC4R (NM_013099). The rat glyceraldehyde-3-phosphate dehydrogenase (GAPDH) gene (NM_017008) and beta-actin (NM_031144) were used as reference genes for data normalization. Relative real-time PCR was performed using real time master mix (Yekta Tajhiz Azma, Tehran, Iran) in a 20 µL volume containing 1 µL cDNA, 1X SYBR Green buffer and 4 pmol of each primer. The amplification reactions were carried out in a StepOne cycler (Applied Biosystems, Foster City, CA, USA) (Table 1). After 40 cycles, the specificity of the amplifications was tested by heating from 60 °C to 95 °C, resulting in melting curves. To ensure that the PCR products were generated from cDNA but not the genomic DNA, proper control reactions were implemented (using RNA as a sample). For quantitative real-time PCR data, the relative expression of the MC4R mRNA was calculated based on the threshold cycle (Ct) method. The Ct for each sample was calculated, using StepOne real-time PCR software (Applied Biosystems, Foster City, CA, USA). Accordingly, the fold expression of the target mRNA over the reference values was calculated by the equation 2^-ΔΔCt^ (14), where ΔCT is determined by subtracting the corresponding internal control Ct value from the specific Ct of the target, MC4R. The ΔΔCt was obtained by subtracting the ΔCt of each experimental sample from that of the calibrator one (castrated male control rats).

**Statistical analysis**

Data on the relative expression of MC4R gene were subjected to the test of normality and analyzed by one-way ANOVA (SPSS for Windows, version 20, SPSS Inc, Chicago, Illinois), and mean separation was performed by post hoc LSD test at P< 0.05.

**Table 1:** Sequences of real-time PCR primers and amplification reactions conditions

**Gene**	**Primers** **5' 3'**	**Amplicon length (bp) **	**Amplification condition**
MC4R	F:GACGGAGGATGCTATGAG R:AGGTTCTTGTTCTTGGCTAT	116	15 min at 94 °C, 40 cycles of 94 °C 10 s, 56.6 °C 15 s, and 72°C 30 s
Beta-actin	F:CCACACTTTCTACAATGAGC R:ATACAGGGACAACACAGC	169	15 min at 94 °C, 40 cycles of 94 °C 15 s, 57.8 °C 20 s, and 72°C 30 s
GAPDH	F:CAAGATGGTGAAGGTCGGTGTG R:CGTGGGTAGAGTCATACTGGAA	158	15 min at 94 °C, 40 cycles of 94 °C 10 s, 60 °C 15 s, and 72°C 30 s

F: forward; R: Reverse

## Results

**Fig. 1:**
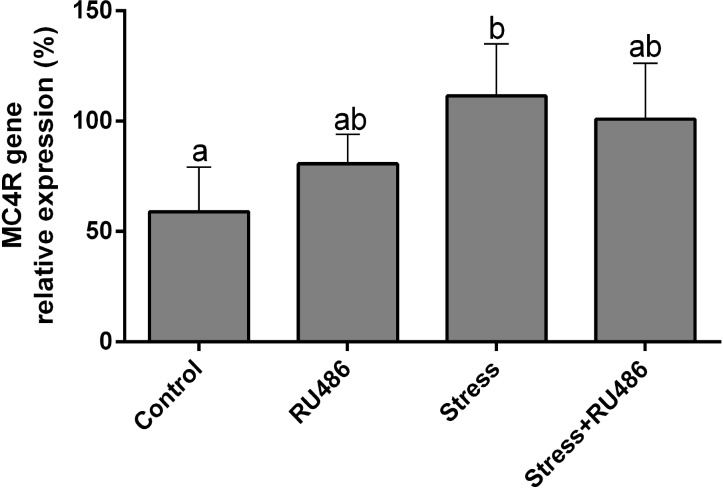
The effect of chronic stress on the relative expression of MC4R mRNA in the hypothalamus of male rats. Values are expressed as mean± SE. ^a,b^ Different superscript letters show significant differences between groups (P< 0.05).

There was no significant difference in weights between rats in all groups (Table 2, P>0.05). Relative expression of MC4R mRNA in ARC in the chronic stress group was higher than that of the control rats (P<0.05, Figure 1). Relative expression of MC4R mRNA was not different between the stress, RU486 and stress/RU486 groups (P> 0.05). Further, no difference was observed in relative expression of MC4R mRNA between the control, RU486, and stress/RU486 groups (P> 0.05).

**Table 2:** Effects of chronic stress on body weight of adult male rats

**Groups**	**Body weight **
Control	226.5± 4.7
RU486	244.8± 7.6
Stress	231.3± 8.0
Stress+ RU486	243.5± 12.6

Values are expressed as mean± SD g

## Discussion

Chronic restraint stress noticeably increased MC4R mRNA expression in ARC of hypothalamus in rats. Consistent with our findings, electrical foot shock stress in rat increased MC4R mRNA in the hypothalamus(15). Moreover, acute stress increased MC4R mRNA in the amygdale of rats (11) and hypothalamus of mice (16). In the present study, this change in increase of MC4R expression after chronic stress was not associated with change in body weight. In contrast, during acute stress in rats decreased MC4R function was observed with increased body weight (17). On the other hand, rapid-eye-movement sleep deprivation stress in rats did not change the expression of MC4R genes in hypothalamus (18). MC4R expressing neurons of the ARC are the POMC neurons, the activation of which reduces food intake behaviors. POMC neurons project from the ARC to the amygdale to mediate anxiety (19). α-MSH production and release are specifically up-regulated due to emotional stress (20). This may reflect changes in element of feeding behavior related to stress signals to a specific neuronal population. Several studies have implicated MC4R in anxiety and stress situations (17, 21) but do not identify the exact mechanism involved. However, it is shown that central administration of a melanocortin agonist increased the effect of chronic stress (22). Moreover, chronic stress had no effect on melanocortin- 4 receptor knock out mice (22)

According to the present findings, relative expression of MC4R mRNA did not change after RU486 injection with or without chronic stress. Chronic stressful condition upregulates MC4R expression and this interaction with stress is not related to glucocorticoid receptor activation. One explanation for the dissociation of MC4R expression and glucocoticoid receptor in stress response in the present study can be explained by the fact that stress exposure is a highly heterogeneous concept with varying levels of timing and severity, and it is not acceptable that stress exposure would exert a uniform effect. Stress likely affects the way the HPA axis and body weight are coupled in different ways depending on the type and time course of stress (23).

In conclusion, it was found that chronic stress caused marked increase in MC4R expression, with no change in this effect in the presence of RU486, a glucocorticoid antagonist.
